# Impaired intratumoral dendritic cell function and potential predictive value of dendritic cell markers for metastasis in malignant salivary gland tumors

**DOI:** 10.4317/medoral.26248

**Published:** 2023-11-22

**Authors:** Ana Guadalupe Gama-Cuellar, Katya Pulido Díaz, Mariana Martínez Calleja, Gabriela Anaya Saavedra, Velia Ramírez-Amador, Jorge Rodarte Corro, Victor Ramón-Ramírez, Ricardo Luiz Albuquerque-Júnior, Rogério Gondak

**Affiliations:** 1 DDS, MSc, PhD. Universidade Federal de Santa Catarina (UFSC), Department of Pathology, Florianópolis, SC, Brazil; 2 DDS, MSc, PhD. Autonomous University of Baja California, School of Health Sciences, Tijuana, Baja California State, Mexico; 3 DDS, MSc, PhD. Autonomous Metropolitan University, Xochimilco Unit, Department of Health Care, Mexico City, State of Mexico, Mexico; 4 MD. Mexican Institute of Social Security, Regional General Hospital No.1, Tijuana, Baja California, Mexico

## Abstract

**Background:**

The differentiation between primary and metastatic salivary gland neoplasms (SGNs) helps in determining appropriate management strategies, including the need for additional diagnostic tests, surveillance, or aggressive treatment. The purpose of this study was to identify and quantify the immature and mature dendritic cells (DCs) in metastatic and no metastatic SGNs and determine its association with clinicopathological findings.

**Material and Methods:**

Cross-sectional, observational, and descriptive study that includes 33 malignant salivary gland neoplasms [MSGN (6, 18.1% metastatic)], and 22 pleomorphic adenomas (PA), as a control group. Clinical and histopathological characteristics were obtained. Immunohistochemistry for human leukocyte antigen D-related (HLA-DR), CD1a, CD83, and Ki-67 proteins was done. Positive intra- and peritumoral DCs were counted.

**Results:**

Individuals with MSGN had a lower density of intratumoral HLA-DR+ cells than those with PA (*p*=0.001), Ki-67 immunostaining was significantly higher in MSGN than in PA (6% vs. 1.4%, *p*<0.001). Metastatic MSGN showed less intratumoral CD1a+ than non-metastatic (3.2 vs. 165.1, *p*=0.001). No differences in intra- and peritumoral CD83+ cells were found between benign and malignant SGN.

**Conclusions:**

These results suggest that the immune-protective function of intratumoral DCs is compromised in MSGNs. DCs markers may represent useful prediction tools for metastases in salivary gland malignancies, with crucial implications in the implementation of appropriate disease management strategies.

## Introduction

Metastatic tumors generally indicate a more advanced stage of the disease and are associated with a worse prognosis, compared to primary malignant tumors (1). Hence, differentiating between metastatic and primary malignant salivary gland neoplasms (MSGN) helps guide clinical decisions since the approach to managing metastatic tumors often involves systemic therapies such as chemotherapy, targeted therapy, or immunotherapy, whereas primary malignant tumors may require local treatment modalities like surgery or radiation therapy (2).

Dendritic cells (DCs) are essential components of the immune system, derived from bone marrow precursors, and are known for their role in activating the immune response and tumor surveillance. In the context of salivary gland neoplasms (SGNs), DCs have been found to infiltrate various tumors influencing the tumor microenvironment and the functions of anti-tumor T-cells (3). The presence of DCs in tumors has been associated with prognosis, where a higher population of DCs is often associated with a better outcome. However, the role of DCs can be complex, as they can have both pro-tumor and anti-tumor effects depending on the specific tumor microenvironment (4,5).

In SGNs and oral squamous cell carcinoma, DCs, along with macrophages and lymphocytes, play a significant role in regulating the immune response within the tumor microenvironment (6-8). The interaction between epithelial and mesenchymal components is crucial for tumor growth, and DCs contribute to this process. Additionally, lymphoid cells can influence areas of high malignancy, contributing to increased cell proliferation in tumors of the parotid gland (8).

The absence of immature DCs, such as Langerhans cells in MSGN suggests a deficiency in antigen presentation, which can promote tumor progression, highlighting the importance of DCs in the antitumoral immune response, underscoring the potential significance of understanding their presence and function in these tumors (6).

Therefore, the objective of this study was to compare the presence of DCs in metastatic and primary MSGN, with a control group consisting of pleomorphic adenomas (PAs). Additionally, the study aimed to investigate the relationship between DC presence and clinical characteristics.

## Material and Methods

Cross-sectional, observational, and descriptive study, conducted following the Helsinki Declaration and approved by the Ethics Committees of the Federal University of Santa Catarina (protocol #09840519.6.0000.0121) and the Autonomous University of Baja California (UABC) (agreement 005/18).

A total of 33 MSGN tissue samples and 22 PAs from two different hospitals, the Regional General Hospital No.1 in Tijuana, Baja California, Mexico, and the Polydoro Ernani de São Thiago University Hospital, in Florianópolis, Santa Catarina, Brazil. The samples were collected between January 2006 and December 2020. Demographic and clinical data, including age, sex, location, tumoral size and tumor node metastasis classification, were obtained from the clinical records.

The tissue samples were fixed in 10% formalin for 24 hours and embedded in paraffin blocks. Sections of 3-μm thickness were obtained from the tissue blocks for histopathological and immunohistochemical analyses. Each sample was analyzed using hematoxylin and eosin staining and based on the histopathological characteristics of both MSGN and PA. The presence of neural invasion, perivascular invasion, necrosis, cystic components, tumor-associated lymphoid proliferation (TALP), and histopathological grade was recorded. Histopathological diagnoses were confirmed by two oral pathologists (R.G. and K.P.D), according to the World Health Organization classification of head and neck tumors (9).

- Immunohistochemistry

Reactions with primary monoclonal mouse antibodies against human leukocyte antigen D-related (HLA-DR; TAL.1B5, 1:50; Dako, California, USA), CD1a (010, 1:100; Dako, California, USA), and CD83 (1H4b, 1:50; Novocastra, Newcastle Upon Tyne, UK) were used to stain the DCs. HLA-DR and CD1a were used for staining immature DCs, whereas CD83 was used for staining mature DCs. Cell proliferation was evaluated using Ki-67 (MIB-1, 1:150; Dako, California, USA). Subsequently, the slides were incubated for 60 min at room temperature with the secondary antibody using the EnVision/Dual-Link System-HRP (K4065, Dako, USA) and developed using a chromogenic substrate (3,3 diaminobenzidine, K3468; DAB, California, USA). The slides were counterstained with Carazzi hematoxylin. For each reaction, negative controls were prepared by omitting the primary antibodies.

Microscopic analysis. Ten fields of each slide were captured at 400x magnification of a binocular optical microscope (Axiostar Plus; Carl Zeiss, Oberkochen, Germany) coupled to a digital image acquisition system (14.3MB; Opticam Camera, Seoul, Korea) and a microcomputer (AOC, Miami, USA), where the images were stored. ImageJ 1.53a software (National Institutes of Health, Maryland, USA) was used to quantify CD1a+, CD83+, and HLA-DR+ cells per area in the intra- and peritumoral compartments (cells/mm2). The Ki-67 proliferation index of the tumor sections was calculated by dividing the number of Ki-67+ cells by the total number of counted cells (1000 cells per case). The labeling site considered positive was the membrane and or cytoplasm for CD1a, CD83, and HLA-DR antibodies. For Ki-67, brown nuclear staining was considered positive.

- Statistical analysis

The descriptive data in this study were summarized using frequencies for categorical variables, while measures of central tendency (mean and median) and dispersion (standard deviation and interquartile range) were calculated for ordinal variables. The association between variables was assessed using various statistical tests, including the Student's t-test, chi-square test, and Fisher's exact test when appropriate. The Mann-Whitney and Kruskal-Wallis tests were utilized for quantitative analysis, followed by Dunn's post hoc test. Spearman's correlation test examined the relationship between the Ki-67 index and immunomarkers. The statistical analysis was conducted using SPSS software version 23.0 (IBM, New York, United States), with a significance level set at 5% (*p*<0.05).

## Results

- Patient population

A total of 33 cases of MSGN and 22 cases of PA, as a control group, were included in the study. Table 1 presents the distribution of sex in both groups, showing an equal representation of males and females. The mean age of the participants was similar in both groups, with an average age in the fifth decade of life. Although the parotid gland was the most commonly affected site in both groups, a significantly higher proportion of malignant salivary gland tumors were observed in minor salivary glands (24.2% vs 4.6%, *p*=0.048). Regarding tumor size, pleomorphic adenomas had a slightly higher median size (3.75 cm) than malignant neoplasms (3 cm); however, this difference was not statistically significant.

Adenoid cystic carcinoma (AdCC) was the most common histopathologic diagnosis in MSGN, observed in 10 patients (30.3%). Mucoepidermoid carcinoma (MEC) was the second most prevalent, detected in 8 cases (24.3%), while acinar cell carcinoma (AcCC) was found in 4 cases (12%). Carcinoma ex pleomorphic adenoma (CAEXPA) and secretory carcinomas were each present in 3 individuals (9.1%). Myoepithelial carcinoma (MeCa) and lymphoepithelial carcinoma (LeCa) accounted for 6.1% of the cases each, while adenocarcinoma not otherwise specified (ACa NOS) comprised 3% of the cases. Regarding MEC, the most frequent histopathologic grade was the intermediate type, observed in four cases (50%), followed by low and high grades, each accounting for 25% of the individuals (2/25% each).

Among the thirty-three individuals with MSGNs, six (18.1%) exhibited metastases. Specifically, four cases (66.6%) showed lung metastases, one case (16.7%) had metastasis in the cervical nodes, and one case (16.7%) had simultaneous involvement of both the lung and cervical nodes. Out of the six cases with metastasis, four (66.6%) were diagnosed as AdCC, one (16.7%) as intermediate-grade MEC, and one (16.7%) as CAEXPA.

Out of the 22 cases of PA, 12 neoplasms (54.5%) were classified as mixed subtypes with myxoid/chondromyxoid features, while 4 individuals (18.2%) exhibited myxoid stroma. Six PAs (27.2%) were characterized as cellular neoplasms without any metaplasia. Among these cellular PA, half of them displayed significant hypercellularity as a noTable characteristic.

Both benign and malignant neoplasms exhibited inflammatory infiltrates, primarily consisting of lymphocytes. These infiltrates were predominantly located in the stroma and displayed a focal distribution. Regarding the characteristics of MSGN, perivascular invasion was observed more frequently (70%) than perineural invasion (48.5%). Additionally, a low proportion of necrosis was identified in 7 out of 33 cases (21.2%).

**Table 1:**
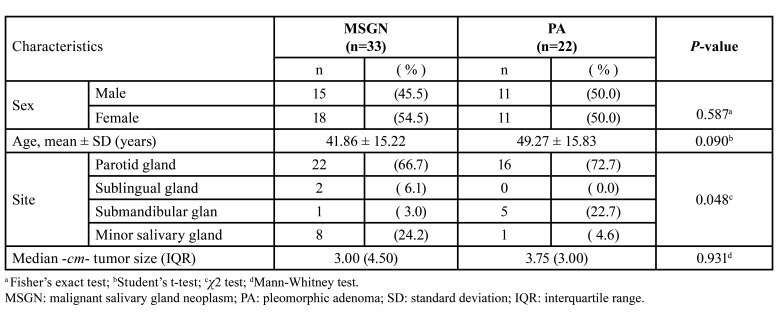
Demographic and clinic characteristics in 55 individuals with salivary gland tumors.

**Figure 1:**
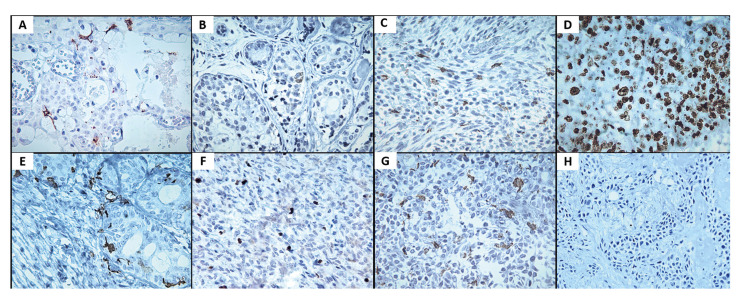
A) Mucoepidermoid carcinoma showing intratumoral immature CD1a+ dendritic cells (DCs). B) Mature CD83+ DCs immunomarked in an adenoid cystic carcinoma. C) Carcinoma ex-pleomorphic adenoma revealing human leukocyte antigen (HLA)-DR+ cells permeating the tumor cells. D) Lymphoepithelial carcinoma revealing high imunoproliferative index (Ki-67). E) CD1a+ DCs with a large number of extensions in pleomorphic adenoma. F) Pleomorphic adenoma showing intratumoral CD83+ DCs. G) High number of HLA-DR+ cells in pleomorphic adenoma. H) Pleomorphic adenoma with scarce immunostaining for Ki-67. Original magnification x 40.

**Figure 2:**
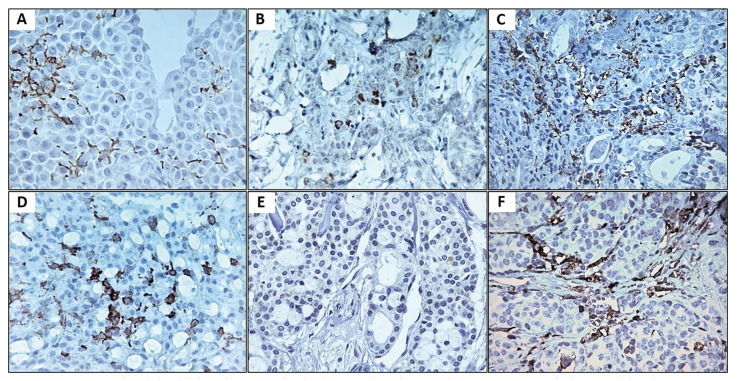
Immunostained dendritic cells (DCs) in the intra and peritumoral compartments of pleomorphic adenoma (PA) and malignant salivary gland neoplasm (MSGN). PA showing CD1a+ DCs. A) and CD83+ DCs. B) located between tumor cells and high concentration of human leukocyte antigen (HLA)-DR+ cells. C) in the peritumoral area. MSGN showing CD1a+ DCs. D) with multiple dendritic extensions and lower concentration of CD83+ DCs. E). Expressive concentration of HLA-DR+ cells around the tumor islands. F). Original magnification x 40.

- Cell proliferation: As anticipated, the median Ki-67 immunoexpression was significantly higher in MSGN than PA (*p*<0.001). Regarding the histological type, no statistically significant differences were identified. There were no statistically significant differences between the different histological types of MSGN. There was no correlation between the Ki-67 index and immunoexpression of CD1a, CD83, and HLA-DR markers. Representative histological samples are shown in Fig. 1.

- CD1a+, CD83+ and HLA-DR+ cells.: The CD1a and CD83 antigens were identified as immature and mature DCs, respectively. Although no significant differences were observed, there was a trend towards a lower quantification of intratumoral CD1a+ and CD83+ cells in MSGN compared to PA (*p*=0.081 and 0.070, respectively). Additionally, a tendency towards a lower number of peritumoral CD1a+ cells was noted in PA compared to MSGN (*p*=0.072). Notably, MSGN with distant metastasis displayed fewer intratumoral CD1a+ cells than those without metastasis (*p*=0.001) (Fig. 2).

MSGN exhibited a significantly lower quantity of intratumoral HLA-DR+ cells than those in the PA group (*p*<0.001). However, the number of cells expressing HLA-DR in the peritumoral compartments was similar between the two groups (*p*=0.993). Additional data regarding these findings can be found in Table 2 and Table 3.

**Table 2:**
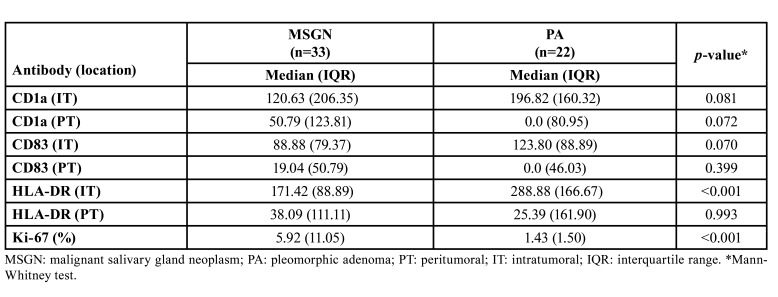
Quantification of CD1a+, CD83+, HLA-DR+ cells (cells/mm2) in the intra and peritumoral compartments of salivary gland neoplasms and Ki-67 index (%).

**Table 3:**
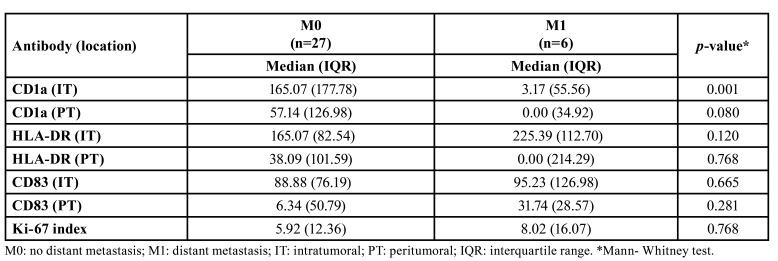
Quantification of CD1a+, CD83+, HLA-DR+ cells (cells/mm2) in the intra and peritumoral compartments of metastatic and non-metastatic malignant salivary gland tumors and Ki-67 index (%).

## Discussion

The results of the present study highlight the role of DCs in the behavior of SGNs. Specifically, the quantification of CD1a+ DCs in MSGN was found to have a significant impact on prognosis. The results revealed that patients with a lower number of intratumoral CD1a+ cells were at a higher risk of developing metastasis. This finding underscores the importance of DC in the progression and metastatic potential of MSGN, suggesting that CD1a+ cell quantification may serve as a valuable prognostic marker in clinical practice.

Several alterations, including acidification, lactate and lipid accumulation, tryptophan deprivation, and hyperglycolysis, can compromise the immune activity of DCs in various tumors, leading to immune escape by the tumor cells (10). These conditions may have diverse clinical implications, including an increased likelihood of metastasis in MSGN.

The current study further confirmed the presence of inflammatory infiltrates as a common characteristic, with a predominance of lymphocytic infiltrates. TALP is more frequently observed in malignant neoplasms, as the metabolism of tumor epithelial cells contributes to the accumulation of lymphoid tissue by producing antigens that stimulate an exaggerated lymphocytic response (11). Additionally, the presence of TALP in early-stage MSGN is associated with a better prognosis and a higher survival rate (8).

The presence, quantity, and maturation status of intra- and peritumoral DCs play a crucial role in the growth regulation of MSGN. Regarding intratumoral DCs, PA tended to exhibit a higher number of immature CD1a+ DCs than the observed in MSGN. Another study by Dultra *et al*. (6) reported a significantly higher proportion of intratumoral CD1a DCs in PA than MSGN (*p*=0.001). This suggests that MSGN neoplastic cells may express inhibitory substances, such as programmed cell death-ligand 1, which reduce the number of DCs and facilitate tumor proliferation and metastasis (12).

Among MSGN, SC and LeCa displayed the highest median number of CD1a+ DCs; however, due to the recent WHO Classification update of head and neck tumors, some cases previously identified as adenocarcinomas or acinar cell carcinomas were reclassified as SC, making a direct comparison with our patients unfeasible. The lymphoproliferative component in LeCa may have facilitated interaction with DCs, potentially contributing to higher median levels of CD1a immunomarkers. Nevertheless, it should be noted that both SC and LeCa are neoplasms capable of producing distant metastases (9).

In a previous study on 12 types of malignant neoplasms (breast, lung, colon and melanoma), including a case of adenoid cystic carcinoma, no immunoexpression of CD83 was reported (13). CD83 is associated with mature DCs, which possess immune regulatory functions and can stimulate and inhibit the immune response against tumor antigens; hence, the immunosuppressive properties of mature DCs may facilitate the growth of MSGN (13).

The quantification of intratumoral HLA-DR+ cells and the Ki-67 tumor proliferation index could serve as additional tools for differentiating between benign and malignant SGNs. In this study, we observed a significant decrease in the median number of cells expressing intratumoral HLA-DR in MSGN compared to benign tumors. While there is currently limited information available on HLA-DR expression in SGNs, previous studies have suggested that low HLA-DR expression may be associated with potential tumor evasion of the immune system by tumors. The evasion can occur through various mechanisms, such as oncogenic changes induced by viruses that result in phenotypic alterations in HLA-DR; the absence of a receptor on natural killer cells that interacts with different HLA molecules, and the presence of heterogeneous cell populations within tumors that generate multiple variants with different HLA phenotypes (14). These mechanisms are relatively rare in benign tumors but can be highly efficient in promoting malignancies (15).

In our cases, Ki-67 immunoexpression was present in a median of 5.9 in MSGN and 1.4 in PA. Specifically, cases of CAEXPA exhibited one of the highest medians for cell proliferation (12.0). These findings are consistent with a Brazilian study that demonstrated significantly higher Ki-67 expression in the carcinomatous areas of CAEXPA compared to PAs (*p* < 0.001), confirming the value of Ki-67 as a cell proliferation marker (16). Similar results were reported by Faur *et al*. (17), who studied 40 cases of SGNs and found that 72% of the cases showed Ki-67 immunoexpression, with CAEXPA exhibiting higher positivity for this marker, supporting the findings of the present study. However, contrasting results were reported in a Danish study, where CAEXPA showed lower median values of Ki-67 staining compared to AdCC, adenocarcinoma NOS, and squamous cell carcinoma (18). Additionally, an Italian study observed Ki-67 immunoexpression in only 9 out of 21 cases of AdCC (19). These varying results suggest that the rate of cell proliferation is not always indicative of tumor grade (20).

Some authors have suggested that Ki-67 values greater than 10% indicate tumor aggressiveness and an unfavourable prognosis (21). The overexpression of Ki-67 supports the carcinogenesis model, meaning a loss of control over cell proliferation due to the accumulation of genetic alterations (22). Therefore, Ki-67 may be a helpful adjuvant diagnostic tool, particularly for SGNs with complex histopathological variations (23). Our study did not find any correlation between the Ki-67 index and the quantification of mature and immature DCs.

The development and progression of cancer involve a highly complex ecosystem consisting of adaptive and innate immune cells. The interaction between these immune cells and the tumor has been associated with various stages of tumorigenesis, including genomic instability, induction of cancer cell proliferation, angiogenesis, and metastasis (24-27). Therefore, understanding the involvement of DCs at different stages of maturation in salivary gland tumors could serve as an important prognostic tool and a potential target for antineoplastic therapies.

## Conclusions

Our findings highlight the potential significance of intratumoral DCs as a crucial defense mechanism against the infiltration of malignant cells. Identifying and quantifying DCs within the intratumoral compartment of SGNs may be a valuable tool for assessing the risk of metastatic spread. By understanding the role of DCs in tumor progression, clinicians and researchers may gain insights into the metastatic potential of MSGNs and develop strategies to enhance the immune response against these malignancies.

## References

[1] Bron LP, Traynor SJ, McNeil EB, O'Brien CJ (2003). Primary and metastatic cancer of the parotid: comparison of clinical behavior in 232 cases. Laryngoscope.

[2] Alfieri S, Granata R, Bergamini C, Resteghini C, Bossi P, Licitra L (2017). F, et al. Systemic therapy in metastatic salivary gland carcinomas: A pathology-driven paradigm?. Oral Oncol.

[3] Devi KS, Anandasabapathy N (2017). The origin of DCs and capacity for immunologic tolerance in central and peripheral tissues. Semin Immunopathol.

[4] Tang M, Diao J, Cattral MS (2017). Molecular mechanisms involved in dendritic cell dysfunction in cancer. Cell Mol Life Sci.

[5] Reichert TE, Scheuer C, Day R, Wagner W, Whiteside TL (2001). The number of intratumoral dendritic cells and zeta-chain expression in T cells as prognostic and survival biomarkers in patients with oral carcinoma. Cancer.

[6] Dultra FK, Barros AC, Schaer-Barbosa H, Figueiredo AL, Gurgel CA, Ramos EA (2012). Immunohistochemical assessment of CD1a-positive Langerhans cells and their relationship with E-cadherin in minor salivary gland tumors. J Oral Pathol Med.

[7] Jardim JF, Gondak R, Galvis MM, Pinto CAL, Kowalski LP (2018). A decreased peritumoral CD1a+ cell number predicts a worse prognosis in oral squamous cell carcinoma. Histopathology.

[8] Velicković LJ, Dimov I, Petrović D, Stojnev S, Dacić S, Velicković S (2013). Stromal reaction and prognosis in acinic cell carcinoma of the salivary gland. Vojnosanit Pregl.

[9] Skálová A, Hyrcza MD, Leivo I (2022). Update from the 5th Edition of the World Health Organization Classification of Head and Neck Tumors: Salivary Glands. Head Neck Pathol.

[10] Peng X, He Y, Huang J, Tao Y, Liu S (2021). Metabolism of Dendritic Cells in Tumor Microenvironment: For Immunotherapy. Front Immunol.

[11] Auclair PL (1994). Tumor-associated lymphoid proliferation in the parotid gland. A potential diagnostic pitfall. Oral Surg Oral Med Oral Pathol.

[12] Harada K, Ferdous T, Ueyama Y (2018). PD-L1 expression in malignant salivary gland tumors. BMC Cancer.

[13] Baleeiro RB, Bergami-Santos PC, Tomiyoshi MY, Gross JL, Haddad F, Pinto CA (2007). Expression of a dendritic cell maturation marker CD83 on tumor cells from lung cancer patients and several human tumor cell lines: is there a biological meaning behind it?. Cancer Immunol Immunother.

[14] Ruiz-Cabello F, Garrido F (1998). HLA and cancer: from research to clinical impact. Immunol Today.

[15] Lei WY, Hsiung SC, Wen SH, Hsieh CH, Chen CL, Wallace CG (2018). Total HLA Class I Antigen Loss with the Downregulation of Antigen-Processing Machinery Components in Two Newly Established Sarcomatoid Hepatocellular Carcinoma Cell Lines. J Immunol Res.

[16] Mariano FV, Costa AF, Gondak RO, Martins AS, Del Negro A, Tincani ÁJ (2015). Cellular Proliferation Index between Carcinoma Ex-Pleomorphic Adenoma and Pleomorphic Adenoma. Braz Dent J.

[17] Faur AC, Sas I, Motoc AG, Cornianu M, Zamfir CL, Lazăr DC (2015). Ki-67 and p53 immunostaining assessment of proliferative activity in salivary tumors. Rom J Morphol Embryol.

[18] Bjørndal K, Krogdahl A, Therkildsen MH, Overgaard J, Johansen J, Kristensen CA (2011). Salivary gland carcinoma in Denmark 1990-2005: a national study of incidence, site and histology. Results of the Danish Head and Neck Cancer Group (DAHANCA). Oral Oncol.

[19] Carlinfante G, Lazzaretti M, Ferrari S, Bianchi B, Crafa P (2005). P53, bcl-2 and Ki-67 expression in adenoid cystic carcinoma of the palate. A clinico-pathologic study of 21 cases with long-term follow-up. Pathol Res Pract.

[20] Larsen SR, Bjørndal K, Godballe C, Krogdahl A (2012). Prognostic significance of Ki-67 in salivary gland carcinomas. J Oral Pathol Med.

[21] Vacchi-Suzzi M, Bocciolini C, Bertarelli C, Dall'Olio D (2010). Ki-67 proliferation rate as a prognostic marker in major salivary gland carcinomas. Ann Otol Rhinol Laryngol.

[22] Jiang YH, Cheng B, Ge MH, Zhang G (2012). The prognostic significance of p63 and Ki-67 expression in myoepithelial carcinoma. Head Neck Oncol.

[23] Díaz KP, Gondak R, Martins LL, de Almeida OP, León JE, Mariano FV (2019). Fatty acid synthase and Ki-67 immunoexpression can be useful for the identification of malignant component in carcinoma ex-pleomorphic adenoma. J Oral Pathol Med.

[24] Gonzalez H, Hagerling C, Werb Z (2018). Roles of the immune system in cancer: from tumor initiation to metastatic progression. Genes Dev.

[25] Mosconi C, de Arruda JAA, de Farias ACR, Oliveira GAQ, de Paula HM, Fonseca FP (2019). Immune microenvironment and evasion mechanisms in adenoid cystic carcinomas of salivary glands. Oral Oncol.

[26] Lavareze L, Scarini JF, de Lima-Souza RA, Emerick C, Sales de Sá R, Aquino IG (2022). Salivary gland cancer in the setting of tumor microenvironment: Translational routes for therapy. Crit Rev Oncol Hematol.

[27] Egal ESA, Scarini JF, de Lima-Souza RA, Lavareze L, Fernandes PM, Emerick C (2022). Tumor microenvironment in salivary gland carcinomas: An orchestrated state of chaos. Oral Oncol.

